# The Medial Sural Artery Perforator Flap versus Other Free Flaps in Head and Neck Reconstruction: A Systematic Review

**DOI:** 10.1055/a-2059-4009

**Published:** 2023-05-29

**Authors:** Yasser Al Omran, Ellie Evans, Chloe Jordan, Tiffanie-Marie Borg, Samar AlOmran, Sarvnaz Sepehripour, Mohammed Ali Akhavani

**Affiliations:** 1 Department of Plastic Surgery, Royal Free National Health Service Foundation Trust, London, United Kingdom; 2 Department of Plastic Surgery, Barts and The London School of Medicine and Dentistry, London, United Kingdom; 3 Academic Plastic Surgery Group, Barts and The London School of Medicine and Dentistry, London, United Kingdom; 4 Department of ENT, Salmaniya Medical Complex, Kingdom of Bahrain

**Keywords:** microsurgery, reconstruction, head and neck

## Abstract

The medial sural artery perforator (MSAP) flap is a versatile fasciocutaneous flap, and yet is less commonly utilized than other free flaps in microvascular reconstructions of the head and neck. The aim is to conduct a high-quality Preferred Reporting Items for Systematic Reviews and Meta-analyses (PRISMA)– and Assessment of Multiple Systematic Reviews 2 (AMSTAR 2)–compliant systematic review comparing the use of the MSAP flap to other microvascular free flaps in the head and neck. Medline, Embase, and Web of Science databases were searched to identify all original comparative studies comparing patients undergoing head and neck reconstruction with an MSAP flap to the radial forearm free flap (RFFF) or anterolateral thigh (ALT) flap from inception to February 2021. Outcome studied were the recipient-site and donor-site morbidities as well as speech and swallow function. A total of 473 articles were identified from title and abstract review. Four studies met the inclusion criteria. Compared with the RFFF and the ALT flaps, the MSAP flap had more recipient-site complications (6.0 vs 10.4%) but less donor-site complications (20.2 vs 7.8%). The MSAP flap demonstrated better overall donor-site appearance and function than the RFFF and ALT flaps ( *p* = 0.0006) but no statistical difference in speech and swallowing function following reconstruction ( *p* = 0.28). Although higher quality studies reviewing the use of the MSAP flap to other free flaps are needed, the MSAP flap provides a viable and effective reconstructive option and should be strongly considered for reconstruction of head and neck defects.

## Introduction

With so few local options available for reconstruction of ablative defects in the head and neck region, microsurgical free tissue transfer remains an imperative tool for the reconstructive surgeon due to their ability to preserve function and appearance. There are various types of vascularized free flaps for the head and neck region, with the choice of flap being dependent on the size and depth of the defect, appropriate tissue composition, quality of donor-site tissue, and surgical preference. Importantly, the choice of vascularized free flap should ideally be performed without leaving the patient with significant donor-site morbidity. For reconstruction of head and neck defects, the free radial forearm flap (RFFF) and the anterolateral thigh (ALT) flap have been employed as the workhorses. 1 2 3 4 The RFFF is widely used because it is a thin flap with a pliable skin paddle, a long vascular pedicle that has a consistent anatomy. However, its use leads to potential significant donor-site morbidity due to potential tendon exposure, scarring, and sacrifice of one of the two major arteries that supplies the distal upper limb. The RFFF is also limited by its size and volume. In contrast, the ALT flap potentially offers greater tissue volume, and patients have generally better donor-site morbidity due to the ability to close the donor site primarily. The limitation of the ALT flap is its bulk, which can be a hindrance when a thin pliable flap is warranted, especially in patients with significantly above-average adipose tissue. 5

**Fig. 1:**
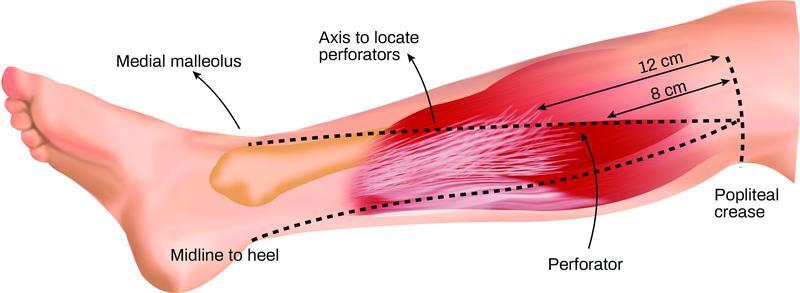
Landmarks to locate the perforators of the medial sural artery perforator flap. The perforators are typically concentrated at an area ∼4.5 cm from the midline and 8–12 cm from the popliteal fossa, along a line drawn from the midline of the popliteal crease down to the medial malleolus.

**Fig. 2:**
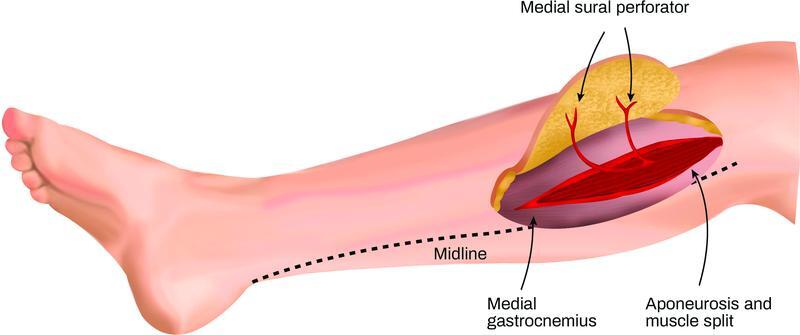
Raised medial sural artery perforator flap.

The medial sural artery perforator (MSAP) flap is becoming increasingly more popular. 4 6 Cavadas et al first described the MSAP flap in 2001 in a series of cadaveric dissections before proceeding to successfully utilizing this flap clinically to reconstruct lower limb and foot soft tissue defects in six patients. 7 The MSAP flap is located in the upper third of the posteromedial calf. The flap usually has two to six perforators that are concentrated in an area 4.5 cm from the midline and 8 to 12 cm from the popliteal fossa crease ( Fig. 1 ), and when harvesting, the pedicled is traced back to popliteal artery by splitting the medial gastrocnemius muscle. The remaining borders of the flap can be incised, and the flap raised ( Fig. 2 ).

The MSAP flap may possess distinct advantages compared with the RFFF and ALT flap in head and neck reconstruction: it is thin, pliable, offers a relatively less hirsute skin paddle, has a long vascular pedicle, and a major artery does not need to be sacrificed when harvesting it. The thinness of the MSAP flap in particular makes it more suitable for floor of mouth and tongue reconstructions than the ALT flap, especially in the Western patient population. 8 9 Additionally, the MSAP flap offers less donor-site morbidity compared with RFFF perhaps owning to its ability to being amenable to direct closure, while the RFFF will nearly always require a split-thickness skin graft at the donor site.

**Fig. 3:**
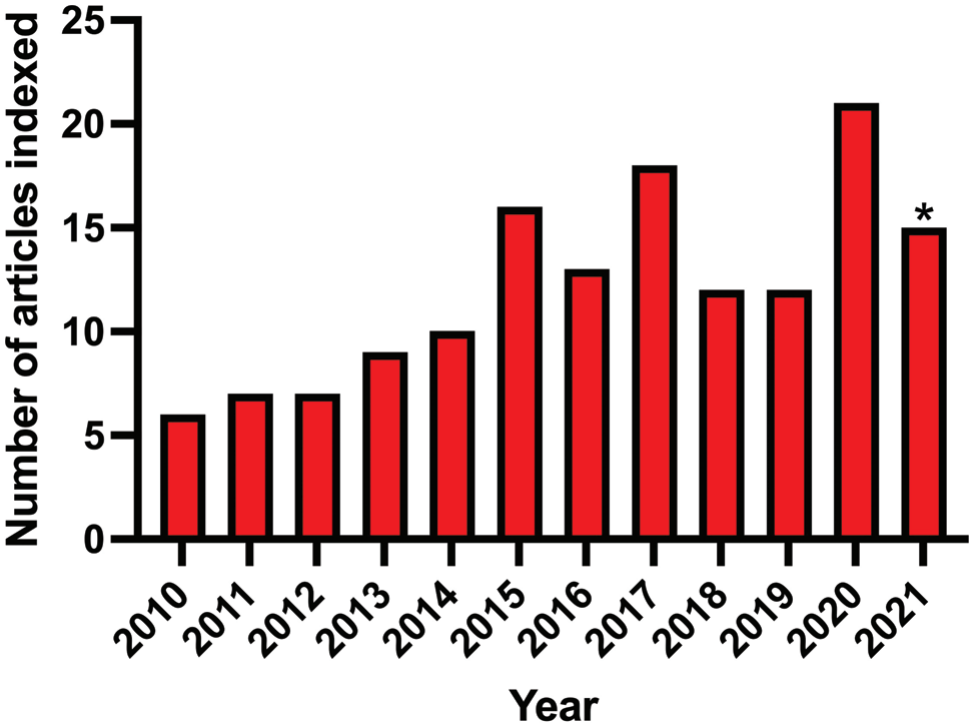
Number of articles published and indexed by Scopus per year under the search term “medial sural artery perforator flap.”

The potential advantages of the MSAP flap compared with the RFFF and ALT flap in head and neck reconstruction have not been fully explored. Most primary studies of the MSAP have been limited in size and scope, mostly presenting small case series at only a singular site. A systematic review and meta-analysis on the MSAP flap was recently reported. However, their review was limited as it “focused on the outcomes of the medial sural artery perforator flap rather than directly comparing it to alternative flaps (e.g., RFFF or ALT flap), which would be a more rigorous study design.” 6 With the increasing number of articles relating to the MSAP flap being published ( Fig. 3 ), a high-quality systematic review that directly compares the MSAP flap with other free flaps in head and neck reconstruction is warranted.

The aim of this study was to perform a comprehensive systematic review of the literature on the MSAP flap compared with other microvascular free flaps (namely, the RFFF and ALT flaps) in patients who had undergone head and neck reconstruction, specifically focusing on functional outcomes, complications, and donor-site morbidity. Given its advantages, the authors hypothesize that the MSAP flap will provide less donor-site morbidity compared with the RFFF and ALT flaps in the context of head and neck reconstruction.

## Methods

This systematic review was conducted in line with “Preferred Reporting Items for Systematic Reviews and Meta-Analyses” (PRISMA) 10 and “Assessment of Multiple Systematic Reviews 2” (AMSTAR 2). 11 Additionally, it was performed in line with the recommendations noted in the Cochrane Handbook for Systematic Reviews, 12 and was without temporal limits. The protocol had been generated a priori and is registered with the unique UIN code: CRD42020205337 on PROSPERO ( https://www.crd.york.ac.uk/PROSPERO ).

Medline, Embase, and Web of Science databases were explored from inception to February 2021. Multiple search strategies, terms, and combination of terms were created and managed using appropriate keywords in English language and was composed of Boolean logical operators. Used search terms included “microsurgery,” “reconstruction,” “plastic surgery,” “MSAP,” “free medial sural artery perforator flap,” “MSAP flap,” “head,” “neck,” “transfer,” “tongue', and “floor of mouth.” The search was not restricted by language and non-English were included beyond title and abstract screening (since the abstract is normally provided in English). All studies involving human participants that primarily aimed to compare a free MSAP flap with either the ALT or RFFF for head and neck reconstruction were included in this systematic review. Therefore, level of evidence 1–3 of the Oxford Center for Evidence-Based Medicine (Randomized Control Trials, Cohort, and Case-Control Studies) were included. 13 Duplicate studies, case reports, studies without original research, studies that reviewed the MSAP flap without a comparator or studies that reviewed MSAP flap without reference to head and neck reconstruction, and animal studies were excluded.

Identified studies were subject to article selection. Article selection occurred in two stages as follows:

1. Titles and abstracts were screened by two teams, composed of two researchers within each team. Disagreements were settled through discussion. When doubt remained about the eligibility, the study proceeded to the next stage.
2. The full text of the articles selected were downloaded and further assessed for inclusion by the two teams. Differences were managed with discussion.

Upon finalization of included studies, data extraction occurred. Data extraction occurred with two teams composed of two researchers within each team. The teams extracted data independently to mitigate the risk of errors. Disagreements were discussed with the supervisor. Elements extracted included author names, countries and year of publication, study design and level of evidence, conflicts of interest and funding, age of participants, number of patients, methodology employed, indication for reconstruction, recipient location, flap characteristics (e.g., flap dimension, size, and thickness), donor-site closure technique, clinical complications, functional outcomes related to speech and oral intake, and donor-site outcomes. To retrieve missing data, the corresponding author of the included journal was contacted via email. If there was no response after 1 week, a further email was sent to ask for missing data. A risk-of-bias assessment was performed using the Risk Of Bias In Non-Randomized Studies – of Intervention (ROBINS-I) tool. 14

Comparable data relating to the donor site of the MSAP flap and other microvascular free flaps were analyzed. As results were mostly presented across a range, patient-reported outcomes data for both MSAP and comparator groups were converted to a binary result of whether patients had acceptable appearance and function of their donor site as well as functional acceptable results of speech and swallowing as noted within the respective studies. For the assessment of donor site, reports of “fair,” “acceptable,” “satisfactory,” or higher among the included studies were combined as overall favorable results, while results of “poor” or “unsatisfactory” were separately combined as unfavorable results. For the assessment of speech and swallowing, reports of “fair,” “acceptable,” “satisfactory” “normal diet” and “normal speech” were combined as favorable results, while results of “poor,” “unsatisfactory,” and “soft or liquid diet” were separately combined as unfavorable results. Review Manager V5.3 (Copenhagen: The Nordic Cochrane Centre, The Cochrane Collaboration. Review Manager [RevMan 5]; Version 5.3; 2014] was used to undertake an assessment of heterogeneity. If a low I ^2^ statistic was noted (I ^2^ < 50%), meta-analysis of available data would have been undertaken using a Mantel–Haenszel fixed-effects model, otherwise a random effects model was utilized. 15 The intention was to perform an additional analysis of characteristics of the MSAP flap compared with other free flaps; however, due to unavailable data, this was not possible.

## Results

**Fig. 4:**
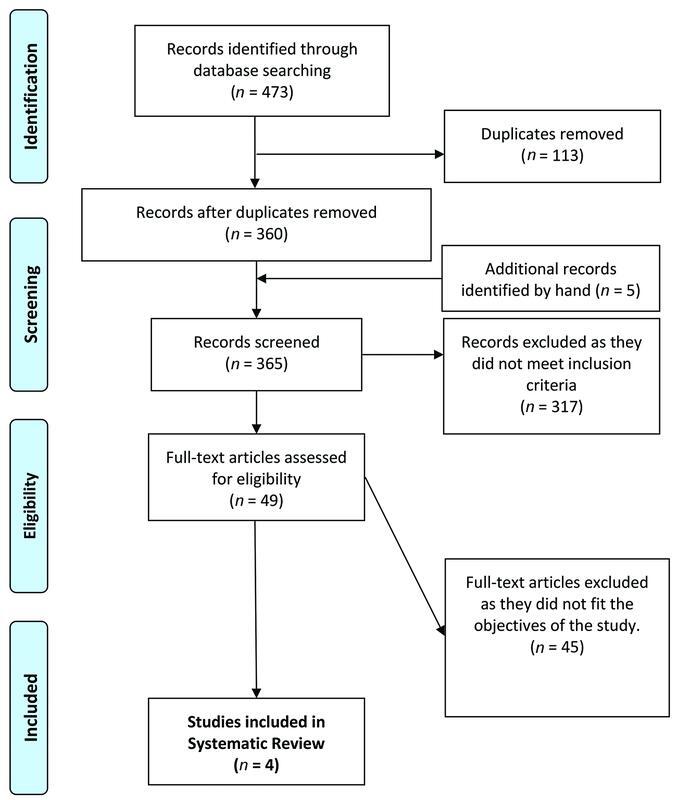
PRISMA diagram showing status of searched articles for review. PRISMA, Preferred Reporting Items for Systematic Reviews and Meta-analyses.

**Fig. 5:**
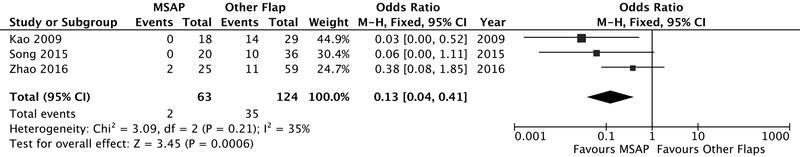
Forrest plot comparing donor-site function and appearance outcomes of the medial sural artery perforator flap vs comparator flaps (radial forearm free flap and anterolateral thigh flap). A Mantel–Haenszel fixed-effects model was used. Odds ratios are shown with 95% confidence intervals.

**Fig. 6:**
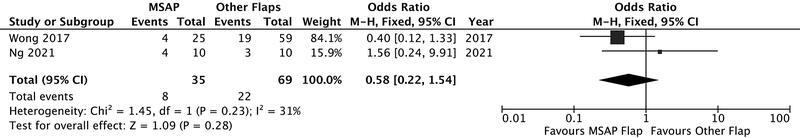
Forrest plot comparing speech and swallowing function outcomes of the medial sural artery perforator flap vs comparator flaps (radial forearm free flap and anterolateral thigh flap). A Mantel–Haenszel fixed-effects model was used. Odds ratios are shown with 95% confidence intervals.

**Fig. 7:**
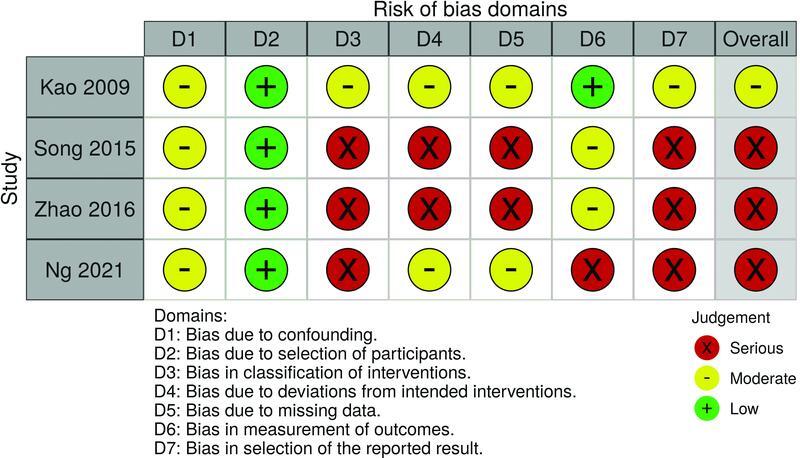
Methodological bias of included studies using the Risk Of Bias in Non-Randomized Studies – of Intervention (ROBINS-I) tool.

A total of 473 articles were identified, of which 4 were included in this systematic review after consensus. ( Fig. 4 ). 16 17 18 19 Articles were excluded if they did not meet the inclusion criteria. In summary, use of the MSAP flap compared with the RFFF or the ALT flap was explored in four studies ( Table 1 ). The 4 included studies consisted of a total of 211 flaps: 77 MSAP flaps, 97 RFFF, and 37 ALT flaps. All studies demonstrated a level of evidence of 3 ( Table 1 ) and were published in English. The MSAP flap had more recipient-site complications but less donor-site complications compared with the ALT or RFFF within the included studies ( Table 2 ). 16 17 18 19 Three of the included studies included patient-reported outcomes of donor-site function and aesthetic appearance for both the MSAP flap and its comparator. 16 17 18 The data were combined and showed a significant statistical difference favoring the MSAP flap compared with the ALT and RFFF flaps in patient-reported donor-site ( *p* = 0.0006; Fig. 5 ). Two of the included studies included patient-reported outcomes of speech and swallowing function for both the MSAP and comparator groups. 18 19 When data were combined, there was no statistical significant difference in speech and swallowing outcomes in patients for head and neck reconstruction within the included studies ( *p* = 0.28; Fig. 6 ). All the included studies were considered at risk of methodological bias ( Fig. 7 ).

**Table 1:** Summary of included studies: patient and flap characteristics

Study		Kao et al (2009) 16	Song et al (2015) 17	Zhao et al (2017) 18	Ng et al (2021) 19
Country		Taiwan	China	China	Singapore
Sample size (flaps)	MSAP	18	24	25	10
	Comparator	29 RFFF	20 RFFF 16 ALT	38 RFFF 21 ALT	10 RFFF
Mean (range) age (y)	MSAP	52.4	59.5 (43–72)	Median = 52 (37–68)	59.1
	Comparator	48.9	NR	NR	60.1
Mean (range) follow-up period (mo)	MSAP	12.5 (3–23)	Mean NR (6–35)	Mean NR (6–24)	NR
	Comparator	23.6 (4–44)	NR	Mean NR (6–24)	NR
Mean (range) harvest time (min)	MSAP	60 (45–90)	NR	65.12 (48–85)	122.5 (100–180)
	Comparator	57.5 (40–75)	NR	RFFF: 58.79 (44–75) ALT: 69.33 (51–92)	75.0 (60–150)
Mean (range) number of perforators used	MSAP	1.7 (1–3)	1.2 (1–2)	2.24 (1–4)	2.0 (1–3)
	Comparator	NR	NR	NR	1.7 (1–2)
Mean (range) pedicle length (cm)	MSAP	Mean NR (9–16)	9.4 (7–13)	10.14 (6.8–12.5)	9.2 (6.0–13.0)
	Comparator	NR	NR	NR	13.2 (10–16)
Mean (range) flap size, cm ^2^	MSAP	67.1 (32–168)	35 (20–80)	NR	59.5 (45–75)
	Comparator	46.1 (18–105)	NR	NR	49.8 (28–84)
Mean (range) flap thickness (mm)	MSAP	NR	5.0 (3.5–8.5)	5.34 (4.1 to 8.7)	7.8 (5–10)
	Comparator	NR	NR	RFF: 5.02 (3.6–7.6) ALT: 8.14 (6.2–11.4)	4.3 (3–6)
Oxford LOE		3	3	3	3
Main findings and conclusions		There was no statistical difference in harvest time, flap size, hospital stay between the MSAP flap and the RFFF. The MSAP flap is a good alternative for head and neck reconstruction of small defects	Use of the MSAP and ALT flaps had significantly higher donor-site aesthetic outcomes than RFF. MSAP is a strong candidate for reconstruction of maxillofacial defects after tumor ablation	No significant difference was found between the MSAP flap and RFFF groups in terms of self-assessed oral comfort, speech, or feeding. However, MSAP flap was found to be better than ALT flap in this respect. Regarding function and cosmesis of the donor-site area, there was no significant difference between the MSAP flap and the ALT flap groups; however, the MSAP flap was better than the RFFF. The MSAP flap is therefore a good alternative for reconstruction after oral carcinoma resection	The MSAP flap is a good option for partial glossectomy reconstruction. The MSAP has similar functional outcomes to the RFF; the main advantage of MSAP when compared with RFF is that it has superior donor-site outcomes. The MSAP can be considered as the near-ideal flap for partial glossectomy reconstruction

Abbreviations: ALT, anterior lateral thigh; LOE, level of evidence; MSAP, medial sural artery perforator; NR, not reported; RFFF, radial forearm free flap.

Note: Values outside of parentheses are presented as the mean unless otherwise. Values in parentheses demonstrate the range.

**Table 2:** Complications of included studies

Study		Kao et al (2009) 16	Song et al (2015) 17	Zhao et al (2017) 18	Ng et al (2021) 19	Total ( *n* )
Recipient-site complication, *n* (%)						
Dehiscence	MSAP	1 of 18 (5.6)	0 (0)	NR	0 (0)	1
	Comparator	4 of 29 (13.8)	NR	NR	1 of 10 (10)	5
Infection	MSAP	NR	0 (0)	NR	NR	0
	Comparator	NR	NR	NR	NR	NR
Fistula	MSAP	1 of 18 (5.6)	0 (0)	NR	NR	1
	Comparator	0 (0)	NR	NR	NR	0
Hematoma	MSAP	0 (0)	0 (0)	NR	NR	0
	Comparator	1 of 29 (3.5)	NR	NR	NR	1
Venous congestion	MSAP	NR	2 of 24 (8.3)	1 of 25 (4)	NR	3
	Comparator	NR	NR	RFFF: 1 of 38 (2.6); ALT: 0 (0)	NR	1
Arterial insufficiency	MSAP	NR	NR	0 (0)	NR	0
	Comparator	NR	NR	0 (0)	NR	0
Partial flap loss	MSAP	0 (0)	1 of 24 (4.2)	0 (0)	1 of 10 (10)	2
	Comparator	1 of 18 (5.6)	NR	0 (0)	0 (0)	1
Flap failure	MSAP	0 (0)	1 of 24 (4.2)	0 (0)	0 (0)	1
	Comparator	0 (0)	NR	0 (0)	0 (0)	0
Other	MSAP	NR	NR	NR	NR	NR
	Comparator	NR	NR	NR	NR	NR
Total recipient-site complication rate	MSAP					8 of 77 (10.4%)
	Comparator					8 of 134 (6.0%)
Donor-site complication, *n* (%)						
Dehiscence	MSAP	NR	0 (0)	1 (4)	NR	1
	Comparator	NR	NR	NR	NR	NR
Altered sensation	MSAP	3 of 18 (6)	NR	NR	0 (0)	3
	Comparator	23 of 29 (79.3)	NR	NR	3 (30)	26 (RFFF)
Delayed healing	MSAP	NR	0 (0)	1 (4)	NR	1
	Comparator	NR	NR	0 (0)	NR	0
Infection	MSAP	NR	NR	NR	1 of 10 (10)	1
	Comparator	NR	NR	NR	0 (0)	0
Hematoma	MSAP	NR	NR	NR	0 (0)	0
	Comparator	NR	NR	NR	1 of 10 (10)	1
Total donor-site complication rate	MSAP					6 of 77 (7.8%)
	Comparator					27 of 134 (20.2%)

Abbreviations: ALT, anterior lateral thigh; MSAP, medial sural artery perforator; NR, not reported; RFFF, radial forearm free flap.

Note: Results are presented as number of flaps. Values in parentheses are percentages.

## Discussion

Flap selection is an important choice to the reconstructive microsurgeon, with the choice being dependent on the defect size and shape, goals of reconstruction, and donor-site morbidity. For years, the RFFF and ALT flaps have been the main workhorse flaps selected by surgeons to reconstruct head and neck defects. However, the MSAP flap is a flap that in principle possesses many distinct advantages for flap selection in head and neck reconstruction: it is a muscle-sparing flap that enables for a versatile flap design, which can be enlarged or reduced in size to fit the recipient site and has relatively low donor-site morbidity and by extension a more satisfied patient and physician. 20 Other systematic reviews have provided an overview of the reported outcomes in the use of the MSAP flap. 6 21 However, these systematic reviews did not provide an assessment of the MSAP flap specific to head and neck reconstruction, nor provide direct comparison of the MSAP flaps to alternative flaps (such as the RFFF or ALT flap), and did provide an assessment of risk of bias. 6 21 The aim of this PRISMA- and AMSTAR 2–compliant systematic review is to assess the use of the MSAP flap compared with the conventionally used RFFF and ALT flaps in head and neck reconstruction and to provide a meta-analysis of comparable data.

## Summary of Study Findings

Four studies were included in this systematic review ( Table 1 ). Kao et al compared 29 RFFF to 18 MSAP flaps in 47 head and neck reconstructions cases. They concluded that the MSAP flap has significantly better donor functional and cosmetic outcomes. 16 Song et al compared 24 MSAP flaps and 20 RFFF for oral and maxillofacial reconstruction using a visual analog scale to assess postoperative oral function and cosmetic results; they concluded that the MSAP achieved significantly higher aesthetic satisfaction at the donor site than with the RFF. 17 Zhao et al used a handheld Doppler to locate MSAP perforators with a 93% detection rate and harvest this flap to reconstruct 25 intraoral defects. They suggested the MSAP to be superior to the RFFF in donor-site appearance and function, and superior to the ALT flap in terms of thickness, postoperative oral sensation, and tongue function. They concluded that the MSAP should be the first choice for reconstruction of soft tissue defects in the oral and maxillofacial region. 18 Ng et al directly compared the MSAP with the RFFF specifically for reconstruction of partial glossectomy defects involving less than 50% of the tongue. They identify that the MSAP and RFF demonstrated comparable functional outcomes and patient satisfaction levels, but patients who had undergone reconstruction with an MSAP flap had fewer donor-site complications compared with those who had reconstruction with RFFF. 19

Details of flap characteristics were accumulated and reported ( Table 1 ). Overall, flap harvest time of the MSAP flap was consistent throughout the studies except for Ng et al who had a significantly longer harvest time compared with the RFFF. 19 The authors of this study were harvesting MSAP and RFFF for defects that were larger compared with the other included studies, which partially explains this difference compared with the other studies. Notably, the authors state that the MSAP flaps also have significantly smaller artery and vein diameters and shorter pedicle lengths compared with the RFFF. 19 However, a direct comparison between the pedicle length and diameters were not reported by the other papers. The average pedicle lengths and ranges for the MSAP flap have been reported previously as 10.1 cm. 6 While this may be less than the average pedicle length for the RFFF and ALT flaps (18 and 12 cm, respectively), 22 23 24 a length of 10 cm has been demonstrated to provide adequate length to anastomose an intraoral flap to the superior thyroid or facial vessels. 25 26

Within the included studies, one or two perforator vessels were harvested and used in the final anastomosis. Anatomical studies have demonstrated two to eight perforators arranged in two vertical rows (a medial and lateral row) along the axis of the leg, with three different branching patters. 27 However, the flap can usually be raised upon one dominant perforator. 27 28 The variation in anatomy coupled with its long intramuscular course can make perforator dissection difficult to alternative flaps, and render the learning curve of MSAP flap harvest to be steep. 26 29 Nevertheless, dissection generally improves with training and experience. 30 Hallock summarized the various methods of perforator identification. 31 Care should be taken when using an audible Doppler as the audio heard can be of the very superficial course of the intramuscular branches and could be confused with the perforator themselves. 31 Alternatively, both color duplex ultrasound and computed tomography angiography have proven instrumental in helping localize the perforators and thereby allowing for swifter harvest of the MSAP flap. 25 32

### Donor-Site Morbidity Analysis

Results from our pooled analysis have shown that the MSAP flap has more recipient-site complications but less donor-site complications compared with the RFFF and ALT flaps ( Table 2 ). Although the included studies report complications, many of the included studies did not fully report their complications and so these findings should be interpreted with caution. A common theme within the included studies is that the MSAP flap leads to less patient donor-site morbidity compared with the RFFF and ALT flaps. This has been statistically significant in some studies 16 17 but not in others. 18 19 This systematic review provided a meta-analysis of donor-site morbidity. Three of the four included studies reported results of a questionnaire that evaluated patient satisfaction with donor-site function and aesthetics for the MSAP flap versus the RFFF and/or the ALT flap. 16 17 18 Results deemed favorable and unfavorable were combined. Findings from our meta-analysis suggest statistically significant overall better patient satisfaction with the MSAP flap versus comparator (RFFF/ALT flaps) in relation to donor-site function and appearance ( Fig. 5 ). The reason for the better donor-site outcomes of the MSAP flap versus the other flaps may be apparent. First, with the RFFF, there is a sacrifice of one major upper limb vessels and there is the possibility of numbness after nerve injury. 33 34 Likewise, the sacrifice of the lateral cutaneous nerve in ALT flaps may be bothersome to patients. 35 As there is less vital structures within the fasciocutaneous harvest of MSAP flap, injury and sacrifice to nerves and vessels are less likely. Second, The RFFF has a conspicuous donor-site location and almost always requires closure with a skin graft, which is then subject to partial graft loss, dyspigmentation, and poor scarring. 33 34 Alternatively, the MSAP flap offers a more inconspicuous donor-site location; the back of the upper calf offers more pliable skin and reports of up to a 5-cm width of the flap can be harvested and the donor site closed directly without the need for skin grafting. 6 18 19 36

### Functional Analysis

For the analysis of speech and swallowing function, two of the four included studies reported speech and swallowing assessments after reconstruction with an MSAP flap versus a comparator. Pooled analysis identified that a trend to improved speech and satisfaction with donor-site function and aesthetics for the MSAP flap versus the RFFF and/or the ALT flap, although this was not statistically significant ( Fig. 6 ). Nevertheless, there are theoretical reasons why the MSAP flap may yield benefit over the RFFF and ALT flaps for intraoral reconstruction. The donor site used to harvest the RFFF may be too thin, and so there is potential lack of bulk of the neotongue, which may be subject to further shrinkage after radiotherapy. 17 37 In contrast, the ALT flap may be too thick, which can cause poor swallow, food retention, and speech unintelligibility that may ultimately necessitate additional debulking. 35 Although a direct comparison within the included studies was not possible due to a lack of reporting, the MSAP flap generally has less subcutaneous fatty tissue than the ALT flap, 8 and may offer the right thickness for partial glossectomy reconstruction. However, the skin of the posterior calf can occasionally be too thick for use in partial glossectomy reconstruction, especially in Western population where the patients may have a higher body mass index, and thus use of alternative flaps may be required. 19 21 38

### Limitations

This systematic review is not without limitations. Unfortunately, all included studies were of evidence level 3. There were also no randomized control trials (RCTs) or prospective studies included in this systematic review, which would have been more robust in methodological design. Equally, using the ROBINS-I tool, three of the included studies were subject to serious bias ( Fig. 7 ). This high risk of bias may affect the validity of our findings. Systematic synthesis and pooled analysis were performed in this study for the subjective assessment of donor site and speech and swallowing as there was enough information to combine data. However, this assumed homogeneity and, by extension, our inference of the findings may be inaccurate. Moreover, this study was hindered by the variable, heterogeneous outcomes reported that prevented systematic synthesis and meta-analysis of findings. The lack of reporting is a limitation that frequently affects systematic reviews. Core outcome sets that standardize reporting have been proposed as a solution in surgical and, specifically, microsurgical studies previously. 39 40 Development of a core outcome dataset may offer a solution to homogenize study findings. 40

## Conclusion

The MSAP flap is a reliable flap for head and neck reconstruction. It is a good alternative for tongue, buccal mucosa, and floor of mouth reconstruction. The advantages of this flap include its thin and pliable skin, good vascular pedicle length, and hairless flap with minimal donor-site morbidity, making it comparable to RAFF and ALT flaps. Evidence from this systematic review suggests that it may possess favorable donor-site appearance and function compared with the RFFF and ALT flaps. However, the quality of evidence of the included studies is poor and is devoid of high-quality RCTs and prospective studies; as a result, included studies may be subject to systemic bias. Further comparison of the MSAP flap to other flaps in the form of high-quality outcome studies and the development of potential core outcome sets that will enable acquisition of heterogeneous data and thus comparison of its use with other flaps are warranted.
